# Single seed precise sowing of maize using computer simulation

**DOI:** 10.1371/journal.pone.0193750

**Published:** 2018-03-05

**Authors:** Longgang Zhao, Zhongzhi Han, Jinzhong Yang, Hua Qi

**Affiliations:** 1 Agronomy College, Shenyang Agricultural University, Shenyang, China; 2 College of Information Science and Engineering, Qingdao Agricultural University, Qingdao, China; 3 College of Agronomy and Plant Protection, Qingdao Agricultural University, Qingdao, China; College of Agricultural Sciences, UNITED STATES

## Abstract

In order to test the feasibility of computer simulation in field maize planting, the selection of the method of single seed precise sowing in maize is studied based on the quadratic function model *Y = A×(D-Dm)*^*2*^*+Ym*, which depicts the relationship between maize yield and planting density. And the advantages and disadvantages of the two planting methods under the condition of single seed sowing are also compared: Method 1 is optimum density planting, while Method 2 is the ideal seedling emergence number planting. It is found that the yield reduction rate and yield fluctuation of Method 2 are all lower than those of Method 1. The yield of Method 2 increased by at least 0.043 t/hm^2^, and showed more advantages over Method 1 with higher yield level. Further study made on the influence of seedling emergence rate on the yield of maize finds that the yields of the two methods are both highly positively correlated with the seedling emergence rate and the standard deviations of their yields are both highly negatively correlated with the seedling emergence rate. For the study of the break-up problem of sparse caused by the method of single seed precise sowing, the definition of seedling missing spots is put forward. The study found that the relationship between number of hundred-dot spot and field seedling emergence rate is as the parabola function *y = -189*.*32x*^*2*^ *+ 309*.*55x - 118*.*95* and the relationship between number of spot missing seedling and field seedling emergence rate is as the negative exponent function *y = 395*.*69e*^*-6*.*144x*^. The results may help to guide the maize seeds production and single seed precise sowing to some extent.

## Introduction

The method of single seed precise sowing is frequently adopted in the process of simplified cultivation of maize, which can greatly reduce the quantity of seeds used and save the trouble of thinning and supplementary planting, thus less labor force is consumed than that of traditional cultivation management[1]. The crop area of maize in China is more than 33 million hm^2^ and the percentage of single seed sowing is less than 40% according to incomplete statistics[2–3]. As more and more migrant workers are moving to work in the cities, study on the simplified cultivation of single seed precise sowing has great significance in reality[4–6]. To find the optimum seed density for maximum yield or maximum profit, breeders carried out a series of studies[7].

The traditional seeding mode is carried out by placing several seeds in one hole to ensure full stand. For example, if the missing seedling rate is required to be less than 1%, then the number of seeds in one hole is≥—2/log(1—field seedling emergence rate). According to the national norm of seeds of 2008[1] that rate of regular seeds’ germination is 85% (assuming the field seedling emergence is equal to the germination rate), the number of seeds n is calculated as n ≥—2/log(1–0.85) = 2.4 and n ≈ 3. Namely, only by placing 3 seeds in one hole, the planting requirement of field missing seedling rate of less than 1% can be met. The missing seedling rate of 1% means the field seedling emergence rate is 99%. In the case of single seed precise sowing, the national norm seems too low. Realizing full stand by single seed sowing seems too far to be true. Today, more and more farmers are adopting the method of single seed sowing, which will soon take the place of the traditional seeding mode. Yet there is not enough theoretical guidance with formidable persuasive power for single seed sowing mode.

**Fig 1:**
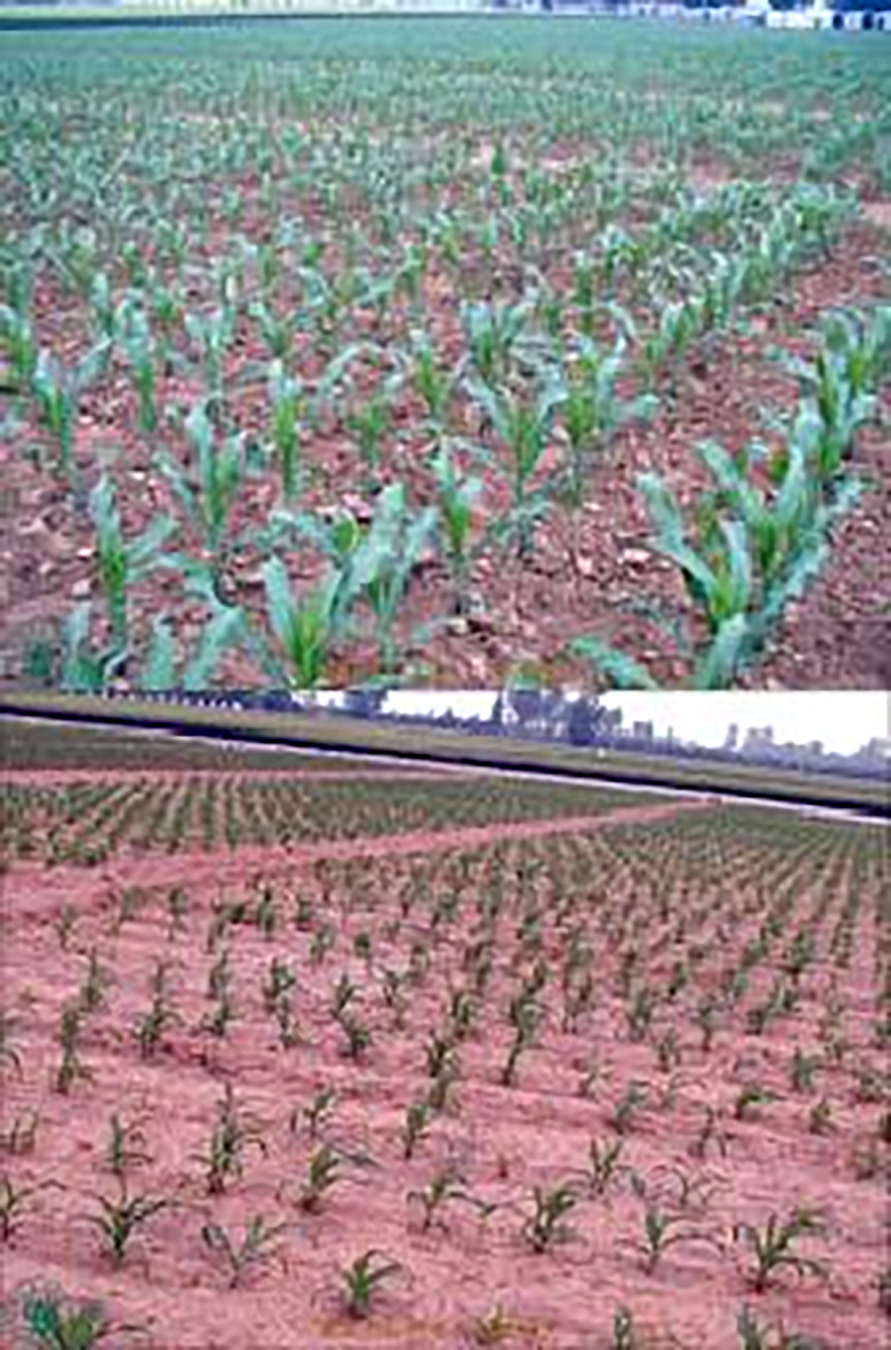
Full and even seedling and sparse breaks up.

Full, even and strong seedling is the foundation of maize prolificacy in Fig 1. While the single seed precise sowing and incomplete seedling emergence can bring about the problem of sparse breaks up, leading to reduction of yield in some degree. Thus in order to get the greatest complex economic benefit, certain field seedling emergence rate must be guaranteed. Then, by adopting the single seed precise sowing, what will the influence of field seedling emergence rate on the yield be like? Different planting methods will influence the yield differently, and then in the case of single seed precise sowing, which planting method is the best? Planting density will influence the yield, then how much will the influence be? Different planting density can lead to the yield compensation because of sparse breaks up, then how much will the compensation be? Traditionally, study and data collection of the above problems are all from field work, which is time consuming and costly. One data collection requires the actual measurement of an entire maize growth period (usually a half to one year), which is too slow and inefficient. As the field experiment is always influenced by factors of the soil, fertilizer, moisture, sunshine and weather etc., the experiment results lack universality. Sometimes each experiment has different results and results of some experiments are even opposite, thus it is difficult to get objective laws. Study results lack universality because of specific field and climate. Moreover, in maize hybridization breeding process, specific breed is always planted in different density empirically to look for its density-tolerance parameter. Thus differences exist in the measurement results of the same field in different years.

Theoretically, whether seeds will germinate is random, so it can be calculated from probability as the field seedling emergence rate. The rows and ridges where maize are planted can be simplified and represented as one grid matrix in computer and the areas of missing seedling are represented as one series connected regions in the computerized binary image. So computer analog simulation can be used to decide on the better method of planting and cultivation under the simple model. With the computer, the field seedling emergence rate, planting density and compensation range of seed yield of planting method can be simulated precisely and the results are precise and objective. Then the results will be further determined by a field experiment and used to enhance the efficiency of maize cultivation study. Based on this consideration, this paper is to find out the advantages and disadvantages of the two methods (Method 1: number of sowing seeds per hm^2^ = recommended planting density; method 2: number of sowing seeds per hm^2^ = recommended planting density/ field seedling emergence rate) which are regularly used in the single seed precise sowing production. Further study is made on the comparison of number and stability of the two methods’ yield, field seedling emergence rate’s influence on the number and stability of yield, missing seedling’s yield compensation mode and the distribution rule of the seedling missing spots (number of hundred-dot spots and number of spot missing seedlings).

## Materials and methods

### Mathematical depiction of the problem

Under the same exterior conditions (such as soil, fertilizer, moisture, sunshine and weather and so on), the planting density of maize may influence its yield. According to the field experiment results of the past years [1, 2], the relationship between maize’s yield and density can be depicted as a singlet curve and curve fitting with quadratic function model can get perfect fitting results [3].

It can be assumed that maize’s yield is the quadratic function of planting density:

$$Y=A\times D^{2}+B\times D+C$$ (1)

In the formula, Y represents yield, D represents planting density. In the parabola model, there are three parameters, namely A, B and C. The two, A and B, respectively the maximum yield and the optimum density embodies explicit biologic and physical meaning. The last one is the quadratic term’s coefficient, which has no explicit biologic and physical meaning but explicit geometric meaning. The vertex formula of quadratic function (1) is Y = A×(D-Dm)^2^+Ym. The vertex P(*Dm*, *Ym*) of the parabola represents the maximum yield that can be acquired at the optimum density with *Dm = -B/2×A* and *Ym = (4×A×C-B*^*2*^*)/4×A*.

According to the vertex formula model(Y = A×(D-Dm)^2^+Ym) of the parabola, yield is the derivative to density:

$$dY/dD=2\times A\times (D-D_{m}),A<0$$ (2)

**Fig 2:**
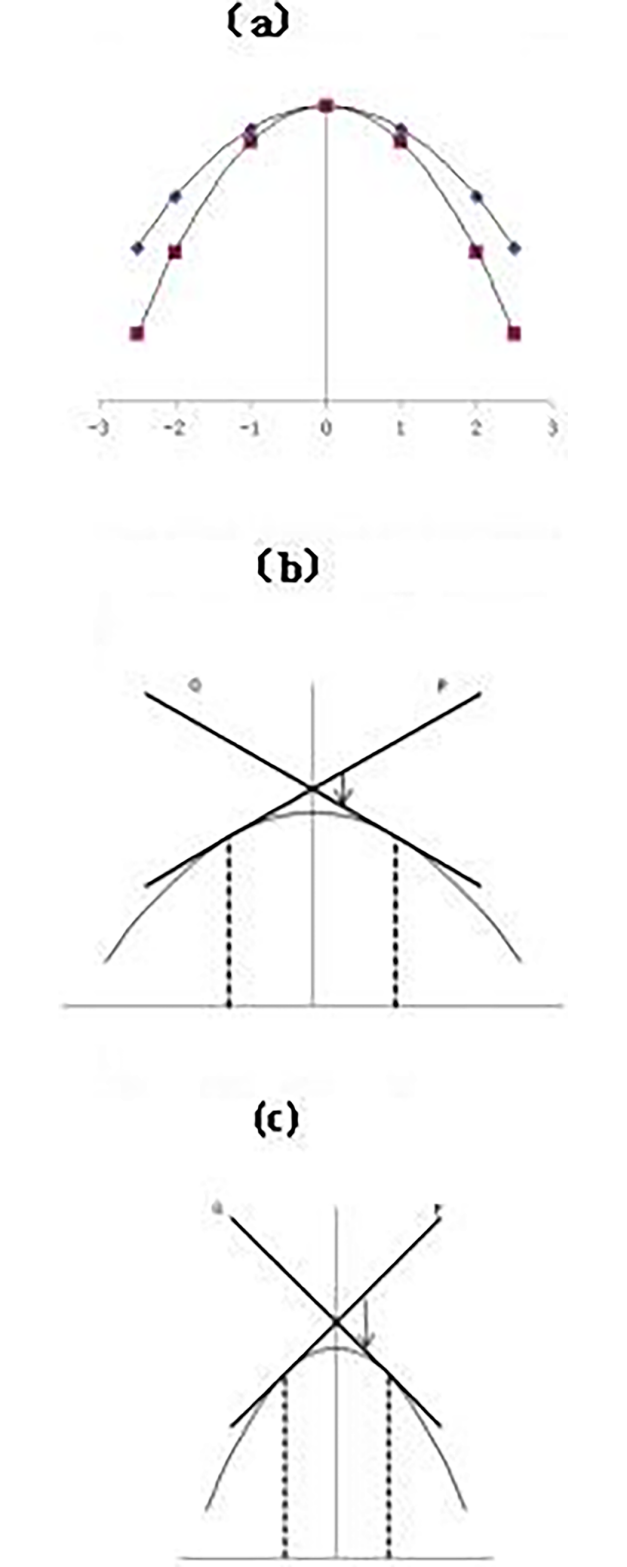
Relationships between yield and planting density. (a)relationship schematic of parabola (b) schema of low sensitivity (c) schema of high sensitivity.

Derivative is the change rate of yield function, which can precisely express the variation of yield brought about by density. When density deviate left or right respectively from the optimum density for one plant/m^2, there is dY/dD = ±2×A. They are the slope of the two tangents which are across the departure point P and Q on the yield curve. Density sensitivity can be defined as the deburrers from tangent P(with the slope>0) to line Q(with the slope<0) showed in Fig 2.

Each variety has an optimum planting density M (individual plant /hm^2^) under certain condition, and the Ms for different varieties vary. Now M is known. Under the ideal state, the number of seedling emergence D*equals M (individual plant /hm^2^). But the actual number of seedling emergence number D^s^ is influenced by *ρ*, which makes the actual number of seedling emergence *D*^*S*^<D^*^. In the case of single seed precise sowing, D^S^ = D* *ρ*. Then:

Method 1: Planting according to the optimum density, the planting number is D*, then the actual number of seedling emergence is *D*^*S*^ = D^*^ *ρ* and the nutrition area of each individual plant is the reciprocal of actual planting number D^*s*^, 1/*D*^*S*^.

Method 2: Planting according to the ideal number of seedling emergence, the planting number is D, then the actual number of seedling emergence is D* and *D*×*ρ* = D*, the nutrition area of each individual plant is the reciprocal of actual planting number D, *1/D*.

Problem: In the case of single seed precise sowing, the two methods, Method 1 and 2, which one is better? And how does the area of missing seedling distribute?

### Method of analog simulation

The simulation platform of the experiment is IBM-SL3000 computer, XP operating system and Matlab2008a software.

#### Computer simulation of the planting method

Seedling emergence methods are automatically generated by computer, and then the random number of 0–1 distribution is generated. 0 represents no seedling emergence and 1 represents seedling emergence. In the binary image, seedling emergence is expressed as black dot while no seedling emergence is expressed as white dot. The generated 0–1 distribution matrix by controlling the emergence probability of 0 and 1, can be used to simulate the seedling emergence status, control the field seedling emergence rate and control the planting number of each hm^2^, namely planting density by controlling the matrix’s rows and columns. This simulation process is realized with the binornd function of Matlab and data are calculated with Excel. The sentence used is R = BINORND(N,P,MM,NN), in which N = 1 represents the random number of 0–1 distribution, 1-p represents field seedling emergence rate, MM and NN represents the planting rows and columns of each hm^2^. Thus MM*NN represents planting strains per hm^2^, i.e. planting density.

**Fig 3:**
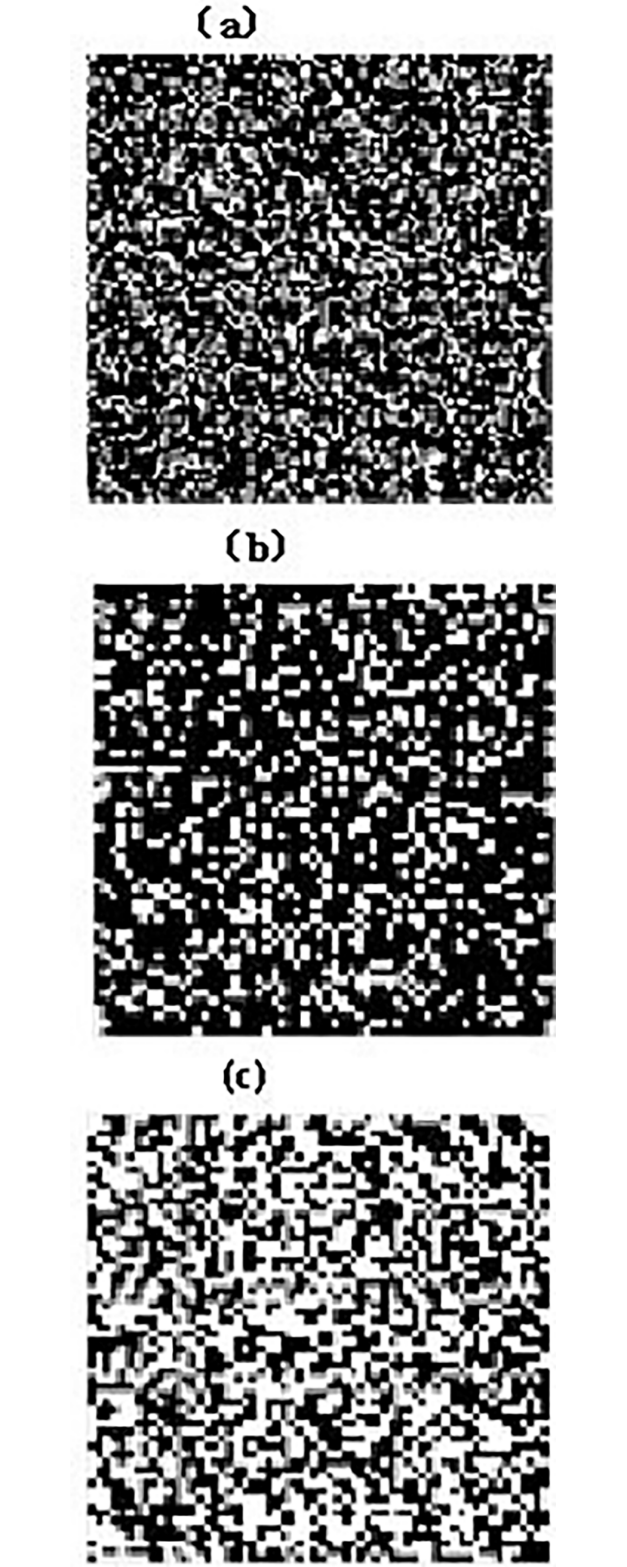
**Simulation images of field seedling emergence under different planting density and field seedling emergence rate (a) 150,000 individual plant /** hm^2^ **and field seedling emergence of 85% (b) 54,000 individual plant /** hm^2^ **and field seedling emergence of 85% (c) 54,000 individual plant /** hm^2^ **and field seedling emergence of 50%**.

Fig 3 shows the simulation images of field seedling emergence in different conditions. They are the seedling emergence status respectively under the condition of 150,000 individual plant / hm^2^ and 54,000 individual plant /hm^2^ with the field emergence rate of 85% and 54,000 individual plant / hm^2^ with the field emergence of 50%.

#### Seedling missing spots and missing seedling compensation

Seedling missing spots is defined as the successive missing seedling area in a successive area. As maize around the missing seedling area has more soil area, growing space and advantage of ventilation and translucidus, there is a certain advantage in the yield of individual plant. This is the so-called yield compensation effect. The compensation effect is related to the floor space of an individual plant. Then how is the floor space determined? Seedling missing spot is shared by maize around it and its area is determined by Voronoi chart [8–11].

Another problem is how to estimate an individual plant’s yield according to its floor space. Many trials have proved that the relationship between maize’s yield and planting density is parabola. The result of the parabola equation divided by planting density is the numeric equation of relationship between an individual plant’s yield and planting density. Planting density and individual plant’s floor space are each other’s reciprocal. Thus this relationship equation can be used to calculate the compensation effect.

**Fig 4:**
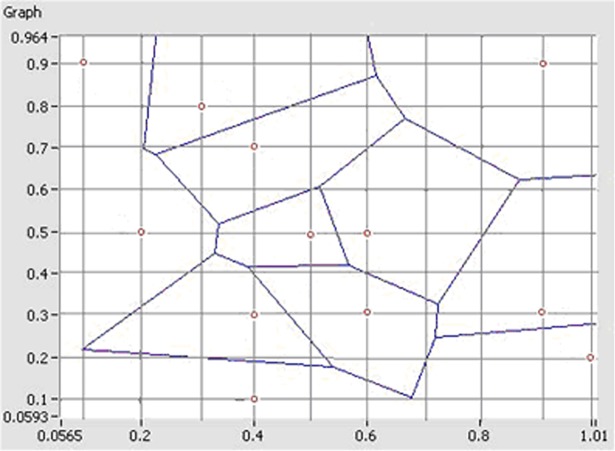
Voronoi diagram. (The red dot indicates lack of seedlings).

According to the parabola model, yield is the parabola function of density, i.e. *Y = F (x)*, then an individual plant’s floor space is the reciprocal of density 1/x, the yield of individual plant is *Yd = F’(1/x)*. The compensation strategy of sprout deficiency: When there is no sprout deficiency, the yield of individual plant should be determined by the planting density, and when there is sprout deficiency, the yield of individual plant should be determined based on the per plant area by Voronoi diagram in Fig 4.

## Result and analysis

### Comparison of yields of the two planting methods

**Fig 5:**
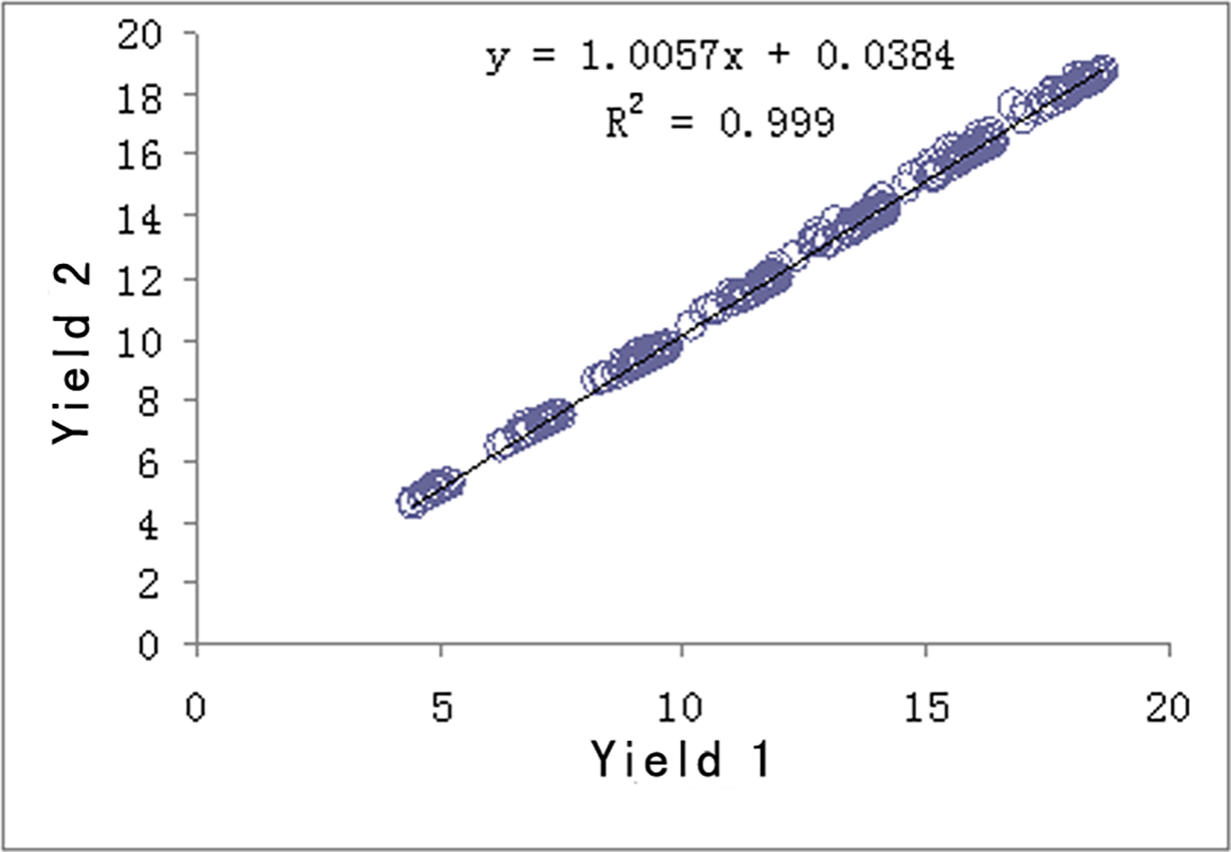
Comparison of yield of two methods.

**Fig 6:**
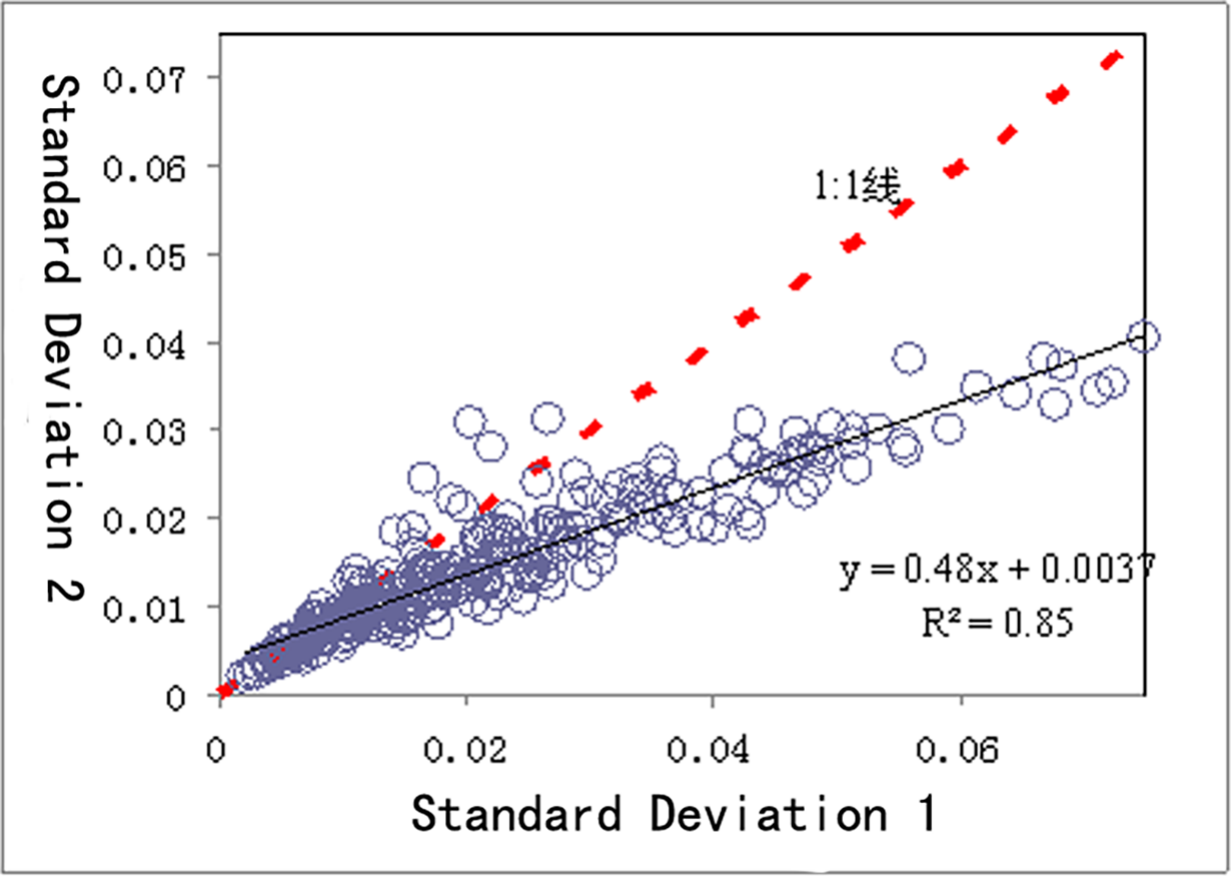
Comparison of two methods’ standard deviation of output.

In the simulation, the row spacing is set at 0.6m, under the condition of optimum planting density being respectively 4.5, 6, 7.5, 9, 10.5, 12 individual plant/m2, planting density is adjusted by changing the planting spacing, which can be calculated according to row space and density. Maximum yield is set at 5.25, 7.5, 9.75,12 and 14.25 ton/hm2 according to different varieties. Under the condition of sensitivity being respectively set at 16,48,80,112 degree and field seedling emergence rate being respectively set at 0.75,0.85,0.9,0.95, relationship between yield and standard deviation of the two methods (statistical data are not listed) are showed in Figs 5 and 6. The setting of above parameters is in line with planting experience [6].

It can be seen from Fig 5 that the yield of Method 2 is always higher than that of Method 1 and the difference is more obvious when the yield is higher. Fig 6 indicates that in terms of deviation of stability of yield, Method 2 is lower than Method 1.

Yield 1 and 2 are both positively related to the seedling emergence rate (both of P are <0.05). The higher the field seedling emergence is, the higher the yield is. Standard deviation 1 and 2 are negatively related to the field seedling rate (both of P are <0.05). The larger the standard deviation is, the lower the stability and the larger the production hazard is. The reason of fluctuation is the change of number of individual plant and field distribution pattern of seedling emergence. The lower the field emergence rate and the larger the change of number of individual plant and field distribution pattern is, the bigger the change of yield is, namely, the larger the standard deviation is. Thus in the production of single seed precise sowing, more attention should be given to the quality of seed, planting bed and seedling quality, and various measures should be adopted to enhance the field seedling emergence rate with a goal to attain high and stable yields.

### Influences of field seedling emergence rate on yields

Table 1 shows the linear regression coefficient of the field seedling emergence rate on the reduction of yields.

**Table 1:** Linear regression coefficient of the field seedling emergence rate on the reduction of yields.

(Optimum density)^a^	Method 1^b^	Method 2^b^
4.5	-5.483	-3.86
6	-10.142	-7.445
7.5	-14.236	-10.912
9	-18.952	-14.875
10.5	-26.118	-20.857
12	-33.604	-27.539

^a^Sensitivity = 112

^b^Results are all similar at random sensitivity

The results indicate that the higher the field seedling emergence rate is, the less the reduction of yield is (the regression coefficient is negative); the reduction speed of yield of Method 2 is always lower than that of Method 1 (the former’s absolute value of regression coefficient is lower than that of the latter).

High field seedling emergence rate is one of the most important conditions of high yield of single seed precise sowing. Under the condition of single seed precise sowing of maize, the method of correspondingly increasing the number of planted seeds based on the field seedling emergence rate to ensure planned number of individual plant is recommended. Method 2 can increase the yield for at least 0.043 t/hm2 and the amount of increased production ascend proportionally with the yield level. Based on the data mentioned above, the yield of Method 2 is higher than that of Method 1.

### Interactions between sensitivity and field seedling emergence rate

Density sensitivity is moderately and positively correlated to the maximum yield [3]. Under different planting density of 4.5, 6, 7.5, 9, 10.5 and 12, the reduction of yield line (maximum yield minus simulated yield), the multiple regression analysis of the two policies’ sensitivity and field seedling emergence rate is showed in Table 2.

**Table 2:** Multiple regression analysis of yield reduction to sensitivity and field seedling emergence rate.

Optimum density	method	Intercept	sensitivity	sensitivity Χrate	R-Square	Maximum yield -simulated yield
4.5	1	-0.033	0.048	-0.049	0.959	0.31
4.5	2	-0.059	0.035	-0.034	0.97	0.238
6	1	-0.011	0.088	-0.091	0.938	0.518
6	2	-0.084	0.067	-0.066	0.97	0.401
7.5	1	0.044	0.123	-0.127	0.958	0.617
7.5	2	-0.047	0.097	-0.097	0.989	0.498
9	1	0.016	0.165	-0.169	0.979	0.584
9	2	-0.044	0.132	-0.133	0.994	0.486
10.5	1	0.122	0.225	-0.233	0.959	0.79
10.5	2	0.004	0.184	-0.186	0.995	0.657
12	1	0.157	0.289	-0.3	0.931	0.888
12	2	0.026	0.242	-0.246	0.991	0.749

When the sensitivity is 112, the multivariate equation is simplified as a linear equation. The common character is the regression coefficients are all negative which indicates that no matter what the values of sensitivity are, the higher the field seedling emergence rate is, the less the yield reduction is.

### Seedling missing spots and its distribution rule

Seedling missing spots is defined as the successive missing seedling area in a successive area which is represented as a region of successive 0 and four series connection region is selected. In the simulation of seedling missing spots under the case of single seed precise sowing, a matrix model of 60 rows and 120 columns is adopted to simulate a piece of land, the simulated row spacing is two times of plant spacing and the field emergence rate is set respectively at 0.95, 0.925, 0.9, 0.85, 0.8, 0.75, 0.7. As the limit of paper length, only the means of 1000 times simulation for each field seedling emergence rate are listed in Table 3.

**Table 3:** Number of missing seedling and the distribution rule of seedling missing spots.

Field seedling emergence rate	Number of seedling missing spots	Number of spot	Number of hundred dot spot	Number of spot missing seedling
0.95	360.8	293.3	4.1	1.2
0.925	541.5	393.4	5.5	1.4
0.9	720.7	465.6	6.5	1.5
0.85	1080.5	538.8	7.5	2
0.8	1438.7	533.4	7.4	2.7
0.75	1799.1	468.7	6.5	3.8
0.7	2159.4	368	5.1	5.9

**Fig 7:**
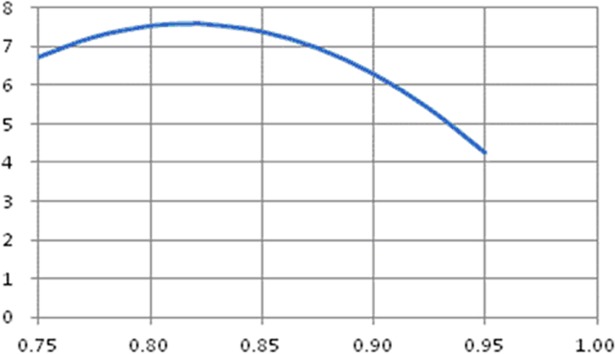
Relationship between number of hundred dot spot and field seedling emergence rate.

Rules acquired from the table are shown as follows:

(1) Number of hundred dot spot is the parabola function of field seedling emergence rate (Fig 7).

$$y=-189.32\;x^{2}+309.55x-118.95$$

Where y is number of hundred-dot spot (i.e. number of seedling missing spots in each 100 planting points), x is field seedling emergence rate. Range of raw data is as x = [0.7,0.95], y = [3.5,8.2]; R^2^ = 0.9499 and extreme point(0.8175,7.5834).

**Fig 8:**
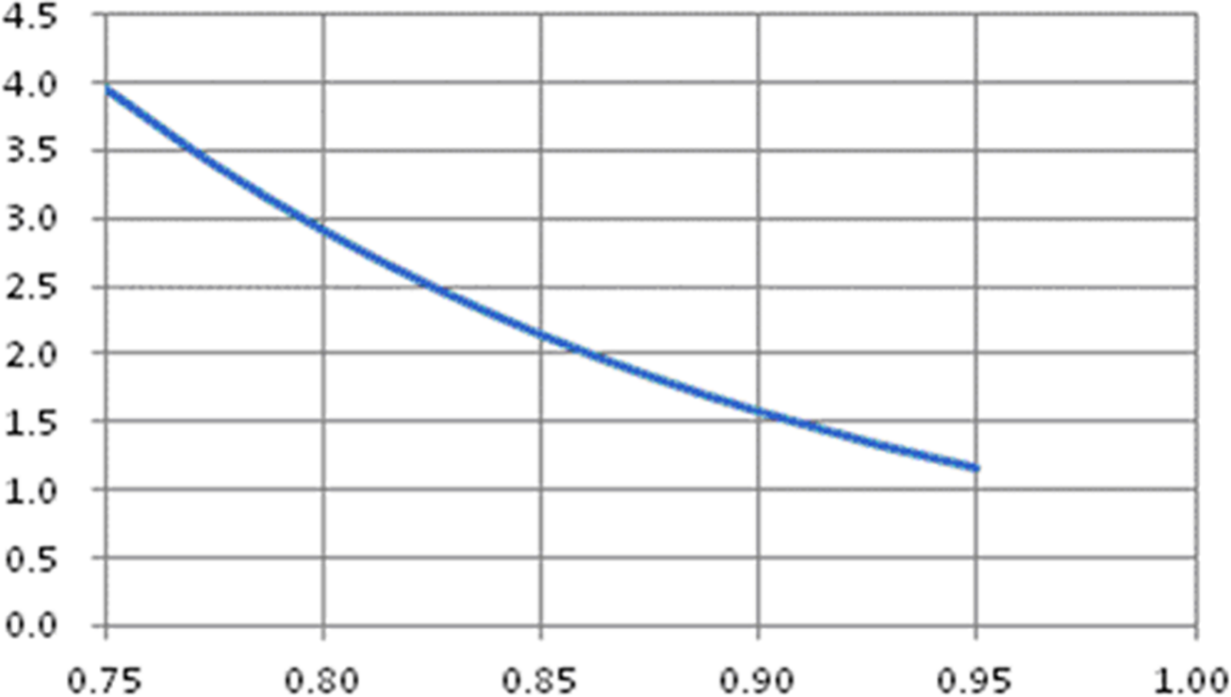
Relationship between number of spot missing seedling and field seedling emergence rate.

(2) Number of spot missing seedling is the negative exponent function of field seedling emergence rate (Fig 8).

$$y=395.69e^{-6.144x}$$

Where y is number of spot missing seedling, namely the number of missing seedling in a seedling missing spots, x is field seedling emergence rate, y = 395.69e-6.144x, R^2^ = 0.9824. Range of raw data is as x = [0.75, 0.95], y = [1.1, 7.6].

Fig 7 shows that when field seedling emergence rate is 0.81, the number of hundred dot spot reaches the summit. When the seedling emergence rate rises or falls, the number of hundred dot spot will always fall, which is different from the instinct. According to the instinct, when the field emergence rate rises, the number of hundred dot spot will fall. But it can be found from the simulation that the number of hundred dot spot is the upper-convex parabola function of field emergence rate. Moreover, the number of spot missing seedling falls with the increase of field seedling emergence rate, but this trend is not linear and the number of spot missing seedling is the negative exponent function of field emergence rate in Fig 8.

The influence factors of maize yield are very complex, It involves variety[12], seed vigor[13], seeding quality and other aspects [14–16]. Planting density is only one of the key factors [17–18]. Our previous studies have shown that Monte-Carlo [19] is an effective simulation method in maize planting.

## Conclusion

In this paper, computer simulation technology is used to simulate field experience, and realize the concept of planting maize in computer. Preliminary study is made on the selection of maize planting density and planting mode. The influence of field seedling emergence rate on maize yield is inspected. Further discussion is made on the problem of sparse breaks up because the field emergence rate can not reach 100% under the case of maize’s single seed precise sowing. The relationship between maize yield model and dominant factor and the definition of seedling missing spots are put forward. The study finds that under the condition of single seed precise sowing of maize, the method of increasing the number of planting seed according to the field seedling emergence rate is recommended to ensure the planned number of individual plants. High field seedling rate is one of the most important conditions of high yield of single seed precise sowing; the number of hundred-dot spot is the upper-convex parabola function of field emergence rate; the number of spot missing seedling is the negative exponent function of field seedling emergence rate. The results have guiding significance to the maize seeds production and single seed precise sowing in some degree.
